# A Review of Recent Developments of Pervaporation Membranes for Ethylene Glycol Purification

**DOI:** 10.3390/membranes12030312

**Published:** 2022-03-10

**Authors:** Valeriia Rostovtseva, Ilya Faykov, Alexandra Pulyalina

**Affiliations:** Institute of Chemistry, Saint Petersburg State University, Universitetskiy pr. 26, 198504 Saint Petersburg, Russia; v.rostovtseva@spbu.ru (V.R.); st022544@student.spbu.ru (I.F.)

**Keywords:** pervaporation, membranes, ethylene glycol, methanol, dehydration

## Abstract

Ethylene glycol (EG) is an essential reagent in the chemical industry including polyester and antifreeze manufacture. In view of the constantly expanding field of EG applications, the search for and implementation of novel economical and environmentally friendly technologies for the separation of organic and aqueous–organic solutions remain an issue. Pervaporation is currently known to significantly reduce the energy and resource consumption of a manufacturer when obtaining high-purity components using automatic, easily scalable, and compact equipment. This review provides an overview of the current research and advances in the pervaporation of EG-containing mixtures (water/EG and methanol/EG), as well as a detailed analysis of the relationship of pervaporation performance with the membrane structure and properties of membrane materials. It is discussed that a controlled change in the structure and transport properties of a membrane is possible using modification methods such as treatment with organic solvents, introduction of nonvolatile additives, polymer blending, crosslinking, and heat treatment. The use of various modifiers is also described, and a particularly positive effect of membrane modification on the separation selectivity is highlighted. Among various polymers, hydrophilic PVA-based membranes stand out for optimal transport properties that they offer for EG dehydrating. Fabricating of TFC membranes with a microporous support layer appears to be a viable approach to the development of productivity without selectivity loss. Special attention is given to the recovery of methanol from EG, including extensive studies of the separation performance of polymer membranes. Membranes based on a CS/PVP blend with inorganic modifiers are specifically promising for methanol removal. With regard to polymer wettability properties, it is worth mentioning that membranes based on hydrophobic polymers (e.g., SPEEK, PBI/PEI, PEC, PPO) are capable of exhibiting much higher selectivity due to diffusion limitations.

## 1. Introduction

**Figure 1:**
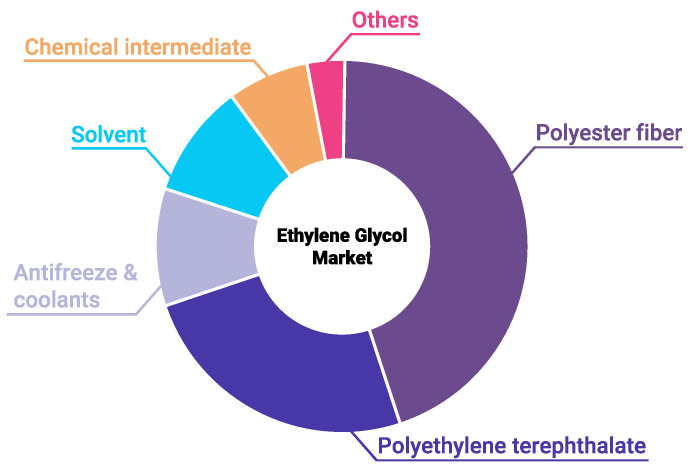
Mono-ethylene glycol market in 2019. Data obtained from Market Research Future.

Ethylene glycol (EG) is an essential reagent in the chemical industry. Among its main applications, worthy of mention are the production of polymers, namely, polyethylene terephthalate and polyester, as well as manufacture of nonvolatile antifreeze and brake fluids, and anti-icing additives [1]. In 2010, the global production and consumption of EG were about 20 million metric tons, with an estimated increase of 5–10% per year [2]. Accordingly, the purification and concentration of waste EG for its regeneration and reuse are of great interest. EG consumption is growing every year, because it is widely used in manufacturing chemicals, in textile and transport industries, and in energy technologies (Figure 1). One of the most important applications of EG is the production of coolants or antifreezes used in cars and personal computers. Due to its low freezing point, EG is also used as an anti-icing fluid for windshields and aircraft. EG is widely employed in the plastics industry to produce polyester fibers and resins, including polyethylene terephthalate (PET).

Recently, EG has been predominantly produced using ethylene oxide derived from petroleum products [3]. However, this process is usually accompanied by high costs and environmental pollution. In addition, excessive amounts of water are consumed to increase EG yield and inhibit the formation of diethylene glycol and triethylene glycol byproducts. Conversely, various nonpetroleum syntheses are gaining widespread occurrence due to the limited resources. For instance, direct synthesis of EG from syngas is one of the most straightforward and most efficient pathways other than petroleum syntheses and allows producing high-purity EG after removing the methanol byproduct from the system [4].

Since EG has a very high boiling point (197 °C), its treatment using traditional methods such as multistage evaporation and distillation is challenging and consumes a lot of energy [5]. Vacuum distillation is also widely used to remove water or methanol. This method requires the presence of additional reagents (e.g., benzene) to create a heterogeneous system with a low boiling point. Such additives are generally toxic and harmful to the environment. Other separation techniques include molecular sieves or adsorption, which are energy-intensive and expensive processes. Therefore, the search for an alternative method for EG dehydration remains a vital issue.

A number of research papers have highlighted the use of pervaporation to remove various impurities from ethylene glycol, which offers great possibilities in obtaining high-purity product [6]. Pervaporation is an environmentally friendly process. The membrane technology is characterized by the high efficiency and long service life of the used membranes. It is expected that realization of this technology will significantly reduce the energy and resource consumption of a manufacturer when obtaining high-purity components using automatic, easily scalable, and compact equipment [7,8,9,10]. Pervaporation is an efficient technique for separation of azeotropic and close-boiling mixtures, since the solution-diffusion mechanism is based not on the relative volatility of the components but on the difference between the sorption and diffusion properties of feed components, as well as on membrane properties [11,12].

Consequently, the choice of a membrane technique is crucial for the separation performance. Today, the main task of membrane research is to develop membrane materials with improved strength-deformation characteristics, as well as high selectivities and permeabilities, which can be produced easily and cost-effectively. There are basically three types of membranes, i.e., inorganic, organic, and mixed matrix (MMM) membranes, which are produced from various membrane materials [13,14,15,16]. A variety of membranes have been investigated for selective water or methanol removal from EG solutions; however, these materials have certain limitations that prevent their industrial-scale production. Hence, the development of advanced membranes with satisfactory separation performance for EG purification still remains an important problem.

This paper provides an overview of the current research and advances in pervaporation of EG mixtures, as well as a detailed analysis of dense polymer membranes, thin-film composites, and mixed matrix materials. Special attention is given to recovery of methanol from EG, including comprehensive studies of the separation performance of polymer membranes. In addition, a brief outline of EG applications, production, and treatment technologies is presented.

## 2. Properties of Ethylene Glycol

EG is the simplest representative of diols. It has a number of unique properties due to its characteristic structure, namely, two hydroxyl (OH) groups at adjacent positions along a hydrocarbon chain.

Table 1 lists various physical properties and constants for EG. It is a colorless, odorless, relatively nonvolatile, and hygroscopic liquid with low viscosity. It tastes sweet and gives a warm sensation to the tongue when swallowing. It is completely miscible with many polar solvents (e.g., water, alcohols, glycol ethers, and acetone) and only slightly soluble in nonpolar solvents such as benzene, toluene, dichloroethane, and chloroform. EG is hard to crystallize. When cooled, it forms a very viscous supercooled mass, which eventually solidifies, forming a glassy substance.

The presence of two hydroxyl groups in the glycol molecule determines its chemical properties and permits reactions such as esterification, dehydration, oxidation, halogenation, and formation of alcoholates and acetals to take place. 

## 3. Pervaporation

**Scheme 1:**
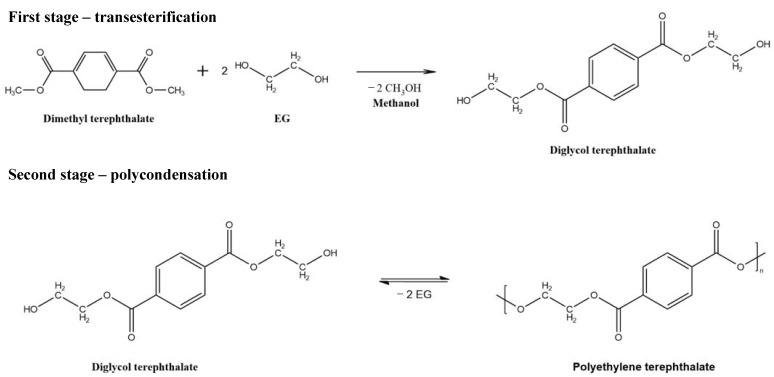
Synthesis of PET.

The formation of methanol/EG and water/EG mixtures is one of the constant stages in the production of EG or PET (Scheme 1); therefore, removing water and methanol from solutions is essential for the regeneration of solvents and the production of ultrapure substances.

Conventional distillation for EG purification has proven to be very expensive as high-pressure steam is required for a reboiler due to the high boiling point of EG despite the absence of azeotrope. EG–water separation by distillation is ranked as the eighth most energy-intensive distillation operation in chemical industries.

Methanol is generated during polyester fiber and PET production, while it must be recovered and regenerated from EG. After esterification, the remaining solution is directed to the methanol recovery system, which separates the methanol by distillation in a methanol column. The vapor from the top of the methanol column is directed to a cold water (or refrigerated) condenser, from which the condensate is returned to the methanol column, and the non-condensable gases are purged with nitrogen before being vented to the atmosphere. The bottom product of the methanol column, mainly EG from the column reboiler, is reused. Depending on the required purity of EG and methanol, the rectification unit can include up to 5–6 columns. Additional purification with the use of chemical reagents is performed to improve the quality of EG.

Membrane methods have shown great potential as efficient and low-cost separation processes. Currently, pervaporation (PV) as a membrane separation method has attracted great attention and covers a wide range of applications, including dehydration of organic media, removal of organic impurities from aqueous solutions, and separation of organic–organic mixtures [18,19]. PV has several significant advantages over traditional separation and purification methods, including high efficiency, low energy consumption, the absence of environmental contamination, and easy scale-up. 

**Figure 2:**
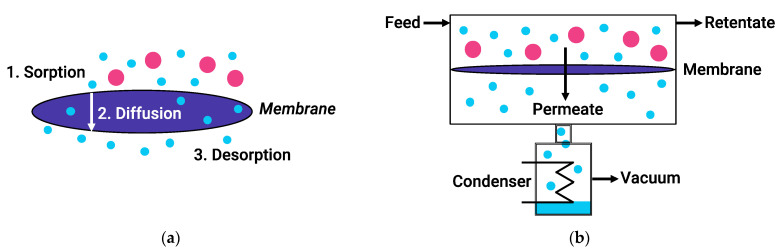
The solution-diffusion model (**a**) and schematic of a typical vacuum pervaporation process (**b**).

During PV, components of a liquid feed mixture penetrate through a membrane and evaporate from its other side, where they form a permeate. In this case, the driving force of the process is the difference in chemical potential between the components of the feed mixture and the permeate. Mass transfer through dense polymer membranes is usually described by the solution-diffusion model (Figure 2). According to this mechanism, the pervaporation process includes three successive stages: sorption/solution of feed molecules at the upstream membrane side, diffusion of the permeate through the membrane, and desorption/evaporation of the permeate into vapor phase at the downstream surface of the membrane. Meanwhile, the sorption and diffusion stages can both reinforce each other and act in the opposite direction, as reflected in the transport characteristics of PV membranes [20]. The main advantage of PV over traditional methods such as distillation is that the PV efficiency is independent of the relative volatility of the components. Therefore, PV not limited by the parameters of vapor–liquid equilibrium [21] and allows separating azeotropic and close-boiling mixtures.

The main transport characteristics of a membrane in the PV process are total flux and the separation factor. These parameters are usually used to compare the performance of different PV membranes. Total flux (*J*) can be determined by calculating the weight of permeate (*M*) formed over time (*t*) with the effective membrane surface area (*S*).

(1) $$J=\frac{M}{S\times t}.$$

The separation factor (*β*) is calculated as follows:

(2) $$\beta =\frac{\left[y/\left(1-y\right)\right]}{\left[x/\left(1-x\right)\right]},$$

where *x* and *y* are the methanol concentrations in the feed and the permeate, respectively.

## 4. Membranes for EG Dehydration

Polymer membranes are the most prominent and frequent choice for pervaporation as they are readily available and exhibit excellent film-forming properties [11,22]. A polymer-based membrane contains crosslinked macromolecular chains that form transport channels facilitating the diffusion of small molecules. Many organic polymers (crystalline, amorphous, glassy, and elastic) are suitable for manufacturing membranes. The choice of material strictly depends on the intended application. Hydrophilic membranes are generally applied in dehydration of liquids, while hydrophobic membranes are used to remove organic compounds from aqueous mixtures. In organic-selective pervaporation (when two or more organic substances must be separated), the choice of membrane is important.

### 4.1. Hydrophilic Membranes

Hydrophilic membranes based on such polymers as polyvinyl alcohol (PVA), cellulose, chitosan, polyimides, and polyacrylonitrile (PAN) are commonly used for PV dehydration of organic solvents. The membrane surface contains high sorption centers that are capable of ion–dipole, dipole–dipole, and hydrogen bonding interactions with water molecules; this provides water selectivity of hydrophilic membranes. In addition, water diffusion is facilitated due to the fact that its molecule is smaller than those of other solvents. Current research involves attempts to vary the composition of the used material and structure of membranes in order to achieve a suitable flux and separation factor. This section is focused on the membrane materials applied for dehydration of ethylene glycol; several pivotal improvements in their pervaporation performance are highlighted.

#### 4.1.1. Poly(vinyl) Alcohol

Poly(vinyl alcohol) (PVA) is a widely used polymer for the pervaporation dehydration of organic solvents due to its high hydrophilicity, easy film-forming ability, and good chemical and mechanical stability [23]. Among the important areas of research involving PVA membranes are modeling and predicting the behavior of the pervaporation process for the case of EG dehydration using thermodynamic equations. Chen and Chen described the results of pervaporation experiment using membranes based on crosslinked PVA [24]. An attempt was made to derive a new equation for the thermodynamic diffusion coefficient considering nonideal solubility and nonideal diffusion effects. The permeate flux values of individual components in water–EG mixtures calculated on the basis of the novel model were in good agreement with the experimental data. The authors also reported the model describing the equilibrium membrane swelling in a liquid mixture as a function of the modified UNIQUAC equation and Flory–Huggins thermodynamics; this swelling is considered one of the stages of pervaporation separation [25]. A good agreement was found between the predictions of the obtained equation and the experimental values for the case of the preferred sorption of water–EG mixtures in a crosslinked membrane. Rezakazemi developed a mathematical model to predict water flows in the pervaporation membrane module [26]. The model was validated using the experimental data obtained during pervaporation through the PVA membrane. The theoretical values were in good agreement with the results of PV experiments. However, the calculated permeate fluxes consistently exceeded the experimentally obtained values. This is due to the fact that the composition of the feed mixture was assumed to be constant. However, water concentration varied in the course of the experiments because of recirculation of the feed phase. Assuming a constant feed concentration, the calculated permeate flux was higher than the experimental value due to the larger concentration difference. The simulation results also showed that permeate flow increased with increasing feed rate and temperature in the membrane module. The membrane material swelled at high feed rates and temperatures; as a result, EG permeated through the membrane, and water concentration on the permeate side was reduced. The developed model was not able to predict this swelling phenomenon.

Despite the active use of PVA as a membrane material, its application is somewhat limited [27]. Due to the presence of a large number of hydroxyl groups (about 38%), PVA is unstable in aqueous solutions, which leads to low selectivity and high fluxes. Several studies were devoted to various methods of PVA modification aimed at increasing efficiency of PV separation of water/EG mixtures.

Kuila et al. performed chemical crosslinking to improve the water selectivity of PVA [28]. Three copolymer membranes with different PVA–copolymer mass ratios (0.25, 0.5, 0.75) were obtained by crosslink polymerization of acrylic acid (AA) and acrylamide (AM) in an aqueous solution of PVA; finally, the polyAAAM copolymer was crosslinked with methylene-*bis*-acrylamide (MBA), and PVA reacted with glutaraldehyde to produce a full interpenetrating network (FIPN) membrane. IPNs are known to have good mechanical strength as compared to membranes prepared by other methods. The effects of membrane composition on selectivity and permeability when separating mixtures containing 1–12 wt.% EG were reported. The low flux can be explained by the high degree of crystallinity of pure PVA due to intra- and intermolecular hydrogen bonding. The inclusion of the copolymer in the PVA matrix increased its hydrophilicity and decreased crystallinity. Thus, an increase in the amount of polyAAAM copolymer led to the increased flux. It was found that the water selectivity of these membranes increased with an increase in the amount of copolymer included in the PVA matrix. However, it was found that FIPN75 showed the highest flux among the three prepared membranes, while its selectivity to water was the lowest, associated with the formation of void space. Meanwhile, the FIPN50 membrane showed optimum performance in terms of both flux and selectivity.

Likewise, Burshe and coworkers investigated a PVA-containing IPN [29]. Along with PVA, polyacrylamide (PAAM) and polyacrylic acid (PAA) were used as hydrophilic membrane materials. However, PAAM and PAA are brittle, and their film-forming properties are unsatisfactory. The PAA–PVA and PAAM–PVA interpenetrating network membranes were obtained by sequential IPN technique. The PAA–PVA IPN membrane containing 30% PAA demonstrated the highest mechanical strength among other membranes used in this study. It was found that the PAA–PVA IPN membrane, unlike the PAAM–PVA IPN film, exhibited selective water transport; this was explained by the fact that the –NH_2_ functional group in PAAM is less hydrophilic than the –COOH group in PAA. Higher values of transport parameters indicated that membrane plasticization occurred under the action of water.

Hyder and Chen provided another method for PVA modification that consisted of mixing a polymer solution with different amounts of chitosan (CS) (25, 50, 70, 75, and 80 wt.%) [30]. It is known that successful mixing can improve separation characteristics, since the mobility of macromolecular chains changes in the process of intermolecular interactions between polymers. Trimesoyl chloride was used to crosslink blended membranes. Good blend compatibility was confirmed by DSC and XRD, while ATR-FTIR confirmed successful crosslinking. The tensile test indicated improved rupture strength of the blended membrane. The results of pervaporation dehydration showed high permeability and selectivity of blended membranes, especially when the amount of chitosan ranged from 70 to 80 wt.%. At 25 °C, the blended membrane containing 75 wt.% chitosan showed good total flux and improved selectivity of 0.11 kg/m^2^·h and 986, respectively. Mixing CS and PVA creates a more compact structure that can preferentially inhibit the diffusion of EG molecules, thereby increasing selectivity.

A membrane based on crosslinked PVA produced by interfacial reaction with glutaraldehyde (GA) was reported in [31]. The required thermomechanical stability and good pervaporation characteristics (for 80 wt.% aqueous solution of EG at 70 °C, the permeability and separation factor were 0.21 kg/m^2^·h and 933) were attained. When the GA content was lower than 15 vol.%, a homogeneous and water-resistant structure was formed on the membrane surface, resulting in increased selectivity. However, when the GA content exceeded 15 vol.%, the resulting material had a compact crosslinked structure and enhanced hydrophobicity; thus, the water selectivity and permeability of this component decreased, which led to a simultaneous decrease in the separation factor and flux. The results of swelling measurements showed that the plasticizing effect of water was the dominant factor causing the abrupt swelling of the PVA (GA15) membrane. In addition, a strong interaction between water and EG was observed, which became more pronounced with increasing water concentration in the feed. This, in turn, effectively inhibited EG penetration and caused a significant increase in the membrane separation selectivity.

Thin-film composite (TFC) membranes based on PVA–polyethersulfone (PVA–PES) were proposed in [32]. PVA–PES composites were synthesized and crosslinked with different amounts (0.2% and 0.5%) of disodium tetraborate (borax). The PVA–PES–0.2% borax membrane turned out to be better than the crosslinked PES–0.5% borax and the uncrosslinked composite in terms of flux and separation factor. The crosslinking composite with borax yielded a membrane with lower crystallinity and improved thermal stability and mechanical properties (as compared to uncrosslinked films). The swelling of the crosslinked composite containing 0.2% borax was less significant than that of the composite crosslinked with 0.5% borax. This was probably due to the fact that addition of a higher amount of crosslinker reduced the free volume of the membrane, which led to restriction of the chain segments’ movement. The resulting PVA–PES composite membrane crosslinked with 0.2% borax showed a separation factor of 3.50 and a total flux of 8.81 m^3^(STP)/(m^2^·h) in the pervaporation of mixture containing 80% EG at 70 °C. Long-term studies of the process conditions showed that the composite membranes exhibited the desired working and structural stability.

In [33], TFC membranes were prepared consisting of an active layer of crosslinked PVA on top of a porous polypropylene (PP) support using glutaraldehyde as a crosslinking agent. The PVA–PP composite membrane with the best characteristics was obtained when the active PVA layer was crosslinked with 0.2 mL GA (50 wt.%). However, when the GA content exceeded 0.2 mL, the increased hydrophobicity and compact crosslinking structure of the membrane caused a reduction in the membrane water selectivity; this led to a simultaneous drop in selectivity and permeability. The membrane was found to have a flux of 0.91 kg/m^2^·h and a separation factor of 1021 for 80 wt.% EG in the feed at 60 °C.

Guo and coauthors produced a TFC consisting of a PES supporting layer and PVA as an active layer [34]. To reduce surface tension and improve the interfacial interaction between the active PVA and supporting layers, the supporting PVA layer was first subjected to interfacial crosslinking with borax. The authors concluded that a rapid reaction between PVA and borax could quickly lead to the formation of a PVA gel network structure and effectively prevent PVA from penetrating the lower support layer. Meanwhile, the addition of a high amount of borax led to the appearance of a thick PVA layer on the PES support layer, which negatively affected the permeation flux and even the selectivity of the composite membrane. The optimal recipe for preparing the PVA/PES composite membrane was as follows: the PES support layer was treated with 0.1 wt.% aqueous borax solution, thoroughly dried, and then immersed into 2 wt.% PVA aqueous solution. The obtained active layer of PVA had a thickness of 1–1.5 µm after double dip-coating. The PVA/PES composite membrane exhibited a high separation factor (over 438), a high permeation flux (0.427 kg/m^2^·h for 80 wt.% EG in the feed at 70 °C), and the desirable structural stability.

Chen and Chen worked on developing a TFC membrane with a dense crosslinked active layer over a PES support membrane [35]. The results showed that membrane performance was determined by both the chemical properties of the membrane material and the physical structure of the membrane. When the crosslinked selective layer is cured at elevated temperatures for comparatively short time, higher temperatures should be used to obtain a more selective membrane. Both the permeation rate and the separation factor increased with an increase in the content of glutaraldehyde used to crosslink PVA due to the inhibition of hydrogen bonds in the PVA membrane. The optimal results (a separation factor of 231 and a flux of 0.383 kg/m^2^·h for the EG concentration in the feed equal to 82.5 wt.% at 80 °C) were obtained for the membranes based on crosslinked PVA containing 3.7 wt.% of the crosslinking agent and 0.05 wt.% of the catalyst.

TFC consisted of a PVA selective layer, and a PSF support was investigated by Hyder et al. [36]. The results showed a consistent trend in the change in physicochemical properties; the degree of crosslinking crystallinity, surface roughness, hydrophobicity, and the degree of swelling diminished with an increase in the concentration of the crosslinking agent trimesoyl chloride (TMS) and the reaction time. The change in TMS concentration influenced the hydrophobicity to a larger extent than variation of the duration of crosslinking reaction with TMS. The experiments involving EG dehydration revealed that the total flux decreased with an increase in the concentration of TMC and the reaction time. The PVA/PSF membrane was found to offer a flux of 0.36 kg/m^2^·h and a separation factor of 987 (90% EG in the feed).

One of the current trends in the field of membrane materials is the development of mixed matrix membranes (MMMs), which can be easily processed, and which have good film-forming properties and high selectivity facilitated by the presence of inorganic particles [37,38]. Highly efficient water-selective membranes for pervaporation dehydration are mainly produced using hydrophilic particles, because they have multiple adsorption sites suitable for water molecules. The membrane operating parameters can also be enhanced by the methods that improve the degree of dispersion of fillers in a polymer matrix, such as use of a softer mixing process, creation of nanoparticles in the polymer matrix in situ, and self-assembly via specific interactions [39,40,41].

Zeolites are crystalline aluminosilicate materials composed of primary elements, including potassium, sodium, magnesium, and calcium [42]. High void volume, elevated surface area, and uniform distribution of pore size led them to be tested as fillers of polymer matrix for membrane-based methods [43]. The introduction of zeolites makes it possible to reduce polymer swelling and enhance the dehydration performance of PV membranes. Baheri et al. used the NaA zeolite to modify PVA-based membranes [44]. The tests confirmed the existence of extended interfaces between two phases and the uniform distribution of the zeolite particles in the polymer matrix. Excellent compatibility of the NaA surface with PVA was attributed to the formation of hydrogen bonds between the zeolite surface (Al–OH and Si–OH) and PVA active surface groups. When the NaA zeolite particles were added to the polymer matrix, the obtained MMM became more hydrophilic. As a result, higher loading of NaA led to higher total permeation flux through PVA–zeolite NaA MMMs. The experimental data showed that addition of the NaA zeolite enhanced both the separation factor and the permeability. The best results were achieved in the experiments involving the membrane loaded with 5 wt.% NaA at 70 °C for 80 wt.% EG feed with a total flux of 2.4 kg/m^2^·h. The separation factor of this MMM was 1520 versus 1202 for the unfilled polymer membrane.

Membranes with a mixed PVA/zeolite NaA matrix were also prepared by Marjani [45]. The zeolites were synthesized by the hydrothermal method. The author concluded that a dense layer of PVA/NaA zeolite membrane with a thickness of about 20 µm was successfully formed on the surface of the mullite substrate. The highest mass transfer flux equal to 0.35 kg/m^2^·h was observed at 60 °C. Shahverdi and coauthors also prepared PVA/zeolite 4A membranes on a polypropylene-based microfiltration support [46]. They found that loading of 5 wt.% 4A nanoparticles into the PVA matrix is optimal for achieving the best separation characteristics. 

Hu et al. fabricated MMMs based on polyvinylamine (PVSM)–PVA modified with carbon nanotubes (CNTs) [47]. The authors showed that the introduction of acid-treated CNTs into the membrane increased the hydrophilicity of the membrane surface. However, no significant change in the solubility of the penetrating substance in the membrane was observed. It was found that the presence of CNTs in the membrane increased its permeability and separation factor, especially for low water concentrations. The PVAm–PVA/CNT (2 wt.%) membrane offered a permeability flux of 0.146 kg/m^2^·h and a separation factor of 1160 (70 °C, water concentration in the feed: 1 wt.%). 

Guo et al. suggested the incorporation of γ-mercaptopropyltrimethoxysilane (MPTMS) into a PVA polymer matrix for the purpose of reducing membrane swelling [48]. It was found that spherical nanosized silica particles were generated and uniformly distributed in the PVA matrix under basic conditions. The formation of covalent and hydrogen bonds between the silica particles and PVA chains increased their compatibility and degree of crosslinking, which resulted in the formation of a more compact structure. Surprisingly, there was no improvement in the pervaporation characteristics of PVA–MPTMS nanocomposite membranes after mercapto groups were oxidized to sulfo groups. The authors reported that, upon inclusion of 50 wt.% MPTMS in PVA, enhanced separation performance was observed with the desired separation factor of 311 and a flux of 0.067 kg/m^2^·h for the 80 wt.% EG mixture at 70 °C.

The same authors also attempted to modify the PVA matrix by joint hydrolysis and co-condensation of γ-glycidyloxypropyltrimethoxysilane (GPTMS) and tetraethoxysilane (TEOS) [49]. They concluded that introducing an appropriate amount of GPTMS effectively prevented the aggregation of silica particles and facilitated a more uniform distribution of these particles in the PVA matrix, thereby greatly optimizing the silica–silica and silica–PVA interfacial interactions. The hybrid membranes with a fixed (GPTMS + TEOS) to PVA mass ratio of 1:1 and a relative molar content of GPTMS equal to 33% demonstrated the highest selectivity (714) with a total flux of 0.060 kg/m^2^·h at 70 °C for 80 wt.% EG in the feed.

Multiple branches of hyperbranched polyester HBPE terminated with hydroxyl groups can improve membrane hydrophilicity, and nanosized voids inside HBPE can increase membrane fluxes. Sun et al. used crosslinked PVA as a basis to prepare MMM filled with HBPE [50]. They synthesized HBPE via a pseudo-step molten-state reaction between 2,2-bis (hydroxymethyl) propionic acid (bis-MPA) and 1,1,1-trimethylolpropane (TMP) using *p*-toluenesulfonic acid (*p*-TSA) as a catalyst. The degree of branching of the resulting HBPE was 0.4551. When the HBPE content exceeded 20 wt.%, formation of particles on the membrane surface was observed, and phase separation between PVA and HBPE occurred. The membrane containing 10 wt.% HBPE had a water flux of 0.044 kg/m^2^·h and a separation factor of 312 for 90 wt.% EG solution at 25 °C. 

Metal–organic frameworks (MOFs) constitute another promising type of filler for MMMs due to their high porosity, the presence of well-defined adsorption sites, and highly customizable properties. However, interfacial defects between the polymer matrix and MOF crystals are inevitable [51]. Zhang et al. proposed to coat MOF (SO3H-MIL-101-Cr) with a thin and uniform layer of polydopamine (PD) to eliminate incompatibility between MOF and the polymer matrix [52]. They reported that the thickness of the PD layer can be effectively controlled by changing the parameters of dopamine self-polymerization, and the presence of this layer improved compatibility between MOF and PVA (due to hydrogen bonding between multiple amino groups of PD and hydroxyl fragments of the PVA matrix). The tested MMM exhibited a significantly improved water permeability of 7.05 × 10^−5^ g/(m·h·kPa).

The pervaporation performance of the polymer membranes reviewed in this section is listed in Table 2. 

#### 4.1.2. Chitosan

Chitosan (CS) or poly(d-glucosamine), a natural biopolymer obtained by deacetylation of chitin, possesses a number of useful characteristics such as hydrophilicity, biocompatibility, antibacterial properties, and remarkable affinity for many substances [53]. CS has been proven to have good film-forming properties; chitosan films demonstrate mechanical and chemical resistance along with high permselectivity for water [54]. CS contains reactive amino and hydroxyl groups that can participate in chemical reactions such as salt formation. These hydrophilic groups play an essential role in preferential water sorption and diffusion through the CS-based membranes. 

A novel method for crosslinking membranes with phosphoric acid in alcohol baths was described by Srinivasa Rao et al. [55]. The crosslinked membrane appeared to have good potential for separation of water/EG mixtures. In pervaporation of the feed containing 90 wt.% EG at 30 °C, a flux of 0.153 kg/m^2^·h and a water concentration in the permeate of more than 93.5 wt.% were achieved. Increasing the membrane thickness led to a drop in the flux value, but the influence of this parameter on separation factor was less pronounced. Higher permeate pressure resulted in lower flow and increased selectivity.

Reddy and coauthors reported membranes based on the mixture of calcium alginate and chitosan (CA/CS) with maleic anhydride (MA) [56]. The experimental results showed that the crosslinked (M-CA/CS) membrane had a good selectivity of 302 at a flux of 0.38 kg/m^2^·h at 30 °C in dehydration of the 96.88 wt.% EG aqueous solution. According to the literature data, the developed membranes had marginally good selectivity and comparable fluxes, making them suitable for dehydration of EG/water mixtures. Moreover, the ease of manufacture of these membranes, combined with their low cost, makes them more attractive for PV processes.

Nam and Lee suggested a novel method for the modification of chitosan composite membranes that consisted of ionic surface crosslinking [57]. Ionic crosslinking allowed the authors to prepare membranes with higher separation factor and lower permeability flux than the uncrosslinked chitosan-based films. The chitosan membranes subjected to crosslinking for 80 min showed the optimal characteristics for dehydration of the EG/water mixtures. At 80 °C and an EG concentration in the feed equal to 80 wt.%, a permeability flux of 1.13 kg/m^2^·h and a water concentration in the permeate of more than 99.5 wt.% were achieved. The effects of operating conditions, including the concentration of the EG in the feed, the operating temperature, and the annealing temperature used for modifying chitosan membranes, on pervaporation characteristics of the chitosan composite membranes were investigated. Annealing of chitosan composite membranes (up to 150 °C) reduced the permeability and separation efficiency of the membranes. A possible reason for these results is an increase in the packing density of the macromolecules after annealing, which led to a decrease in permeability.

Polyelectrolyte complex membranes (PECMs) consisting of CS and PAA were prepared by mixing two solutions of polymers with different ratios [58]. It has been gradually recognized that the preparation of PECMs effectively enhances the pervaporation performance of chitosan. The formed polyelectrolyte complexes had increased charge density and exhibited higher affinity toward water molecules than the pure chitosan membranes. Mechanical tests showed that there was the optimal content of chitosan in the membrane (50%) that provided effective improvement of its mechanical properties. The flux decreased with an increase in the PAAc content in the polyelectrolyte complex, reached a minimum when the PAAc content was 40 wt.%, and then began to increase. It was shown that a higher PAAc content in complex membranes led to more compact packing of the chitosan chains, thus reducing the available free volume of the membrane and decreasing the permeability flux. Moreover, when excess PAAc was added, the PAAc chains that could not form ionic bonds with chitosan moved more freely than the ionically bound polymers. The polymer networks became relaxed and disordered; as a result, the free volume of the membrane and its permeability increased, while the separation coefficient decreased. Among all the considered membranes, PECM60/40 had the highest separation factor of 105 and a flux of 0.22 kg/m^2^·h in the pervaporation of 80 wt.% aqueous solution of EG at 70 °C. 

Hydrophilic mordenite (a zeolite mineral) is widely used in membrane applications. Mordenite has an average pore size of about 0.4 nm, which is larger than the kinetic diameter of a water molecule (0.26 nm), but smaller than that of an EG molecule (0.45 nm). Therefore, Hu et al. used it for modifying the complex membranes of chitosan and poly(acrylic acid) and PECM [59]. They found that the permeability flux decreased, while the separation factor first increased and then decreased with increasing mordenite content. The authors attributed these results to the fact that mordenite reduces mobility of polymer chains and occupies the cavity through which the components penetrate; hence, both of these factors would cause a drop in membrane permeability. M04-PECM60/40 containing 4 wt.% mordenite showed the optimal separation characteristics when dehydrating 80 wt.% EG mixture at 70 °C (a separation factor of 258 with a total flux of 0.165 kg/m^2^·h). 

Dogan and Hilmioglu proposed to improve the structure of zeolite-filled regenerated cellulose membrane (specifically, to overcome the undesirable leakage of ethylene glycol molecules from the membrane) by coating its surface with chitosan (which is chemically similar to cellulose). Regenerated cellulose membranes containing different amounts of zeolite showed low separation factors, while chitosan-coated zeolite-filled cellulose films demonstrated a higher separation factor because coating of zeolite-filled membrane with chitosan eliminated the nonselective voids [60]. 

The pervaporation performance of the chitosan membranes in EG dehydration reviewed in this section can be found in Table 3.

### 4.2. Miscellaneous Polymers

Several other polymers (e.g., perfluoropolymers, polyether ketones, polybenzimidazole, polyimides, and polyamides) have also been reported to show great potential in EG dehydration. Since diffusion is one of the key factors in high-water permselectivity, a number of studies were performed to reveal the effect of membrane structure on transport properties.

Tang et al. adopted a perfluoropolymer membrane known as CMS-3 to separate aqueous mixtures of high- and low-volatility solvents [61]. Despite its high hydrophobicity, the size of the void volume of the material allows small molecules of solvents such as water to pass relatively easily as vapor. Separation factors ranging from 2420 to 12,800 were observed as the EG content of the feed increased from 70 wt.% to 95 wt.%, while the flux decreased from 0.041 to 0.0025 kg/m^2^·h as the concentration of water increased from 10 wt.% to 30 wt.%. It was found that the separation factor of the PDD–TFE membrane was very high at the low feed temperatures when compared to several other membranes. The fluxes were somewhat low, and the used membranes were relatively thick.

Polymers with intrinsic microporosity (PIM) are attractive materials for membrane separation processes. Chen et al. developed carboxylated PIM-1 (cPIM-1) membranes with an adjustable degree of carboxylation up to 0.94 [62]. Carboxylation created many small diffusion channels and significantly enhanced the hydrophilicity of the membrane, which increased linearly with the degree of carboxylation. The authors showed that the hydrophilicity of the membrane had a minimal effect on its selectivity due to the similar interaction of the carboxyl group with both water and EG, as indicated by the almost identical degree of sorption for all the membranes. However, the d-distance of polymers, which reduces hydrophilicity, promoted the diffusion of water molecules rather than EG molecules, which led to a sharp increase in diffusion selectivity with an increase in the degree of carboxylation. This explained why the separation factor increased linearly with the hydrophilicity of the cPIM-1 membranes. Compared to other membranes, the cPIM-1 membranes exhibited relatively high flux values. Typically, the cPIM-1 membrane with a degree of carboxylation of 0.94 achieved a total flux of 13.68 kg·μm/m^2^·h and a separation factor of 75.92 in the pervaporation of an 80 wt.% aqueous solution of EG at 30 °C.

Huang et al. carried out pervaporation experiments using membranes made of sulfonated polyether ketone SPEEK [6]. During the penetration process, relaxation of the membrane was observed, which led to a decrease in the penetration rate. It was found that heat treatment facilitated the relaxation process. However, further relaxation in the heat-treated (3 h at 80 °C) membrane was possible during penetration of feed due to the plasticizing effect of water molecules on the membrane matrix. On the contrary, the EG effect could be neglected. In the case of dehydration of EG aqueous solutions, the process of membrane relaxation ended after almost 3 h of penetration. The SPEEK membranes demonstrated both preferential sorption and preferential transport for water molecules. The penetration of particles can be explained by the modified solution-diffusion mechanism. The water distribution coefficient was obtained using sorption experiments, which decreased with an increase in the initial water content.

Composite membranes with an integrated selective layer were prepared using the “fusion” technique, in which the selective layer of the composite membrane was prepared in advance [63]. The SPEEK top layer was found to be partially dissolved and perfectly integrated into the substrate due to the formation of the SPEEK/substrate material mixture. The structural stability of the composite membrane was analyzed in terms of the properties of a ternary blend layer, which also acted as a permeation barrier layer. The superior pervaporation separation characteristics of the composite membrane demonstrated in EG dehydration were attributed to the suppression of surface layer swelling through effective substrate reinforcement. 

Dual-layer polybenzimidazole/polyetherimide (PBI/PEI) hollow-fiber membranes were developed and applied in for EG dehydration by pervaporation [64]. Three types of membranes were prepared: (1) flat dense PBI membranes, (2) single-layer PBI hollow-fiber membranes, and (3) dual-layer PBI/PEI hollow-fiber membranes. The flat dense PBI membranes had the lowest separation efficiency due to strong swelling. The single-layer PBI hollow-fiber membranes exhibited superior separation performance in terms of permeability and separation factor but had very low tensile strain values. The dual-layer PBI/PEI hollow-fiber membranes had better separation performance due to (1) the unique combination of superior physicochemical properties of the PBI selective layer and relatively low swelling degree of the PEI backing layer, and (2) the synergistic effect of molecularly developed membrane (which was obtained by dual-layer co-extrusion). The authors found the optimum spinning parameters (an air gap of 2 cm and a low take-up speed of 4.60 m/min) for the production of membranes with the highest separation capacity. Thermal treatment of dual-layer PBI/PEI hollow-fiber membranes at moderate temperature (75 °C) allowed the authors to improve separation efficiency. PBI-D-C had a separation factor of 4500 with a comparable high flux (0.186 kg/m^2^·h) for the dehydration of the feed solution containing 64 wt.% EG.

Wang et al. continued investigations of PBI/PEI hollow-fiber dual-layer membranes and performed pervaporation experiments under various operating conditions [65]. The experimental results showed that an increase in operating temperature caused an increase in flux and selectivity, but permeability and separation factors were reduced. Both flux and permeability decreased with increasing permeate pressure, while separation factor and selectivity tended to increase and decrease, respectively. An increase in the EG concentration in the feed from 50 to 90 wt.% led to a decline in the water flux and permeability, but the EG flux and permeability first grew and then decreased. This was due to the combined effect of water-induced membrane swelling and formation of the EG boundary layer on the membrane surface. Dual-layer hollow-fiber membranes with good long-term stability were obtained (they maintained their operating parameters for at least 33 days in the pervaporation separation of 80 wt.% EG aqueous solution at 60 °C).

In [66], a poly(ether-*block*-amide) (Pebax 2533) membrane was synthesized on a superporous polyvinylidene fluoride (PVDF) support. The Pebax 2533 membrane demonstrated the necessary potential for dehydration alcohols, showing a selectivity of 978 with a flux of 0.05 kg/m^2^·h for the feed mixture containing 95 wt.% EG. The authors found that the membrane could be successfully applied as a thin-film composite material to remove small amounts of water (<10%). To optimize flux and bring it closer to the literature values, the authors proposed to cast a thinner Pebax layer (2 µm or less) onto the PVDF substrate.

Polyelectrolytes are promising materials for solvent dehydration membranes due to their superior hydrophilicity. Electrostatic self-assembly of oppositely charged polyelectrolytes provides a versatile and environmentally friendly method for making thin-film composite membranes with ultrathin shell layers. Xu et al. developed a TFC via layer-by-layer self-assembly of polyelectrolytes on an interfacially polymerized polyamide membrane deposited on a microporous polysulfone substrate [67]. Polyethyleneimine and poly(acrylic acid) were used as polycations and polyanions, respectively. The use of the interfacially polymerized polyamide membrane as a substrate could significantly reduce the number of polyelectrolyte bilayers required to form a permeable selective pervaporation membrane. The authors found that increasing the number of bilayers promoted growth in membrane selectivity, but the rate of selectivity increase was reduced after casting two double layers. Use of the membrane consisting of three bilayers allowed the authors to achieve a flux of 0.4 kg/m^2^·h and a separation factor of 340 (40 °C, water concentration in the feed equal to 3 wt.%).

Zhang et al. also investigated polyelectrolyte membranes prepared by layer-by-layer self-assembly over an interfacial polymerized polyamide substrate [68]. It was found that composite membranes based on polyethyleneimine/poly(acrylic acid) (PEIm/PAA) complexes were suitable for EG dehydration, but had unsatisfactory characteristics for ethanol and isopropyl alcohol dehydration at relatively high feed concentrations of the alcohols. The authors explained this result by instability of the PEIm/PAA bilayers and the polyamide support in the solvents. The improvement of membrane selectivity was achieved by replacing PEIm with partially protonated chitosan in the last few polyelectrolyte bilayers during membrane fabrication. It was demonstrated that polyelectrolyte membranes for EG dehydration containing fewer than eight bilayers could be fabricated using an interface polymerized polyamide membrane as a substrate.

Among various preparation methods for composite membranes, interfacial polymerization and crosslinking have attracted special attention. During interfacial polymerization, a crosslinked polymer layer is formed at the interface between two reacting phases (i.e., immiscible liquid–liquid phases, vapor–liquid phases, or solid–liquid phases). The crosslinked layer acts as a barrier against the reacting particles, which causes the formation of an asymmetric structure consisting of a tightly crosslinked outer surface and a less crosslinked inner surface. While the outer surface layer provides selectivity, the less crosslinked part provides high permeability (since the polymer chain mobility in this layer is higher). Du and coauthors [69] prepared a TFC membrane of poly(*N*,*N*-dimethylaminoethyl methacrylate)/polysulfone (PDMAEMA/PSF) by solid/liquid interfacial crosslinking. It is interesting to note that the crosslinked polymer was insoluble in water, but the surface hydrophilicity increased after crosslinking due to the formation of the quaternary ammonium salt. When water concentration in the feed was equal to 1 mol.%, a flux of ~1 mol/m^2^·h and a water permeate concentration of 99.7 mol.% were achieved at 30 °C. This result favorably distinguishes PDMAEMA/PSF from other membranes operating under similar conditions.

Wu et al. studied the effect of the number and sequence of sublayers in the skin layer of the membrane consisting of polyamide (PA) and polydopamine (PD) on pervaporation performance of this membrane [70]. They showed that the presence of one or two PD sublayers as a surface layer (i.e., on top of PA) or a transition layer between PA and substrate facilitated an increase in both the permeability flux and the selectivity of EG dehydration. The [PD]–[PA]–[PD] membrane showed a total flux of 0.081 kg/m^2^·h and a separation factor of 390 at 38°C, when the feed contained 2.4 wt.% of water. The presence of inorganic salt (NaCl) in the feed solution reduced the permeability of EG more significantly than that of water, which led to an increase in the separation factor for EG dehydration.

A membrane based on graphene oxide (GO)-incorporated polyelectrolyte complex nanoparticles (PEC NPs) was developed by Wu et al. [71]. The GO-containing PEC nanoparticles suitable for solution processing were prepared using a new “complex precipitation–redispersion” strategy. It was found that the membrane not only had an increased fractional free volume, but also contained additional cavities between the particles, thus exhibiting significantly improved permeate flux. The authors reported that the spherical PEC NP building blocks provided additional cavities and, therefore, increased membrane flux, while the tightly coupled ion pair structure combined with strong interface interactions between regularly spaced GO nanosheets and the PEC matrix resulted in high selectivity. The prepared PEC NPM/GO-3 membrane achieved a flux of 0.961 kg/m^2^·h and water content in the permeate 99.25 wt.% with a 90 wt.% EG mixture at 60 °C. 

Halakoo and Feng produced membranes by layer-by-layer self-assembly of polyethyleneimine (PEI) and graphene oxide (GO) on a chlorine-treated thin-film composite polyamide substrate [72]. The authors studied the influence of the number of PEI/GO bilayers on membrane separation performance. The membrane surface turned out to be not sufficiently selective for pervaporation separation if the surface “defects” were not completely covered by the first several bilayers. The separation factor increased by 148% as the number of PEI/GO bilayers increased from one to 15 due to a 38% decrease in total flux. The PEI/GO with three bilayers was found to achieve a permeability flux of 0.1 kg/m^2^·h.

**Figure 3:**
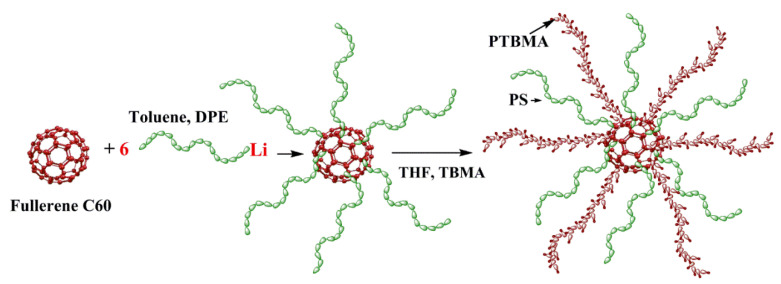
The synthesis of heteroarm star (HAS), where DPE is 1,1-diphenylethylene, THF is tetrahydrofuran, and TBMA is *tert*-butyl methacrylate [76].

Introducing star-shaped macromolecules as fillers can be a promising way to improve the separation capacity of pervaporation membranes [73,74,75]. Hybrid membranes based on poly(2,6-dimethyl-1,4-phenylene oxide) (PPO) modified with heteroarm stars (HAS) (Figure 3) were developed by Rostovtseva et al. [76]. HAS consists of a small C_60_ fullerene branching center and 12 arms of different nature: six arms of nonpolar polystyrene and six arms of polar poly-*tert*-butyl methacrylate. It was reported that the inclusion of HAS led to a change in the structure of the membrane due to the disturbance of the initial packing of polymer chains. Thus, the presence of this star-shaped polymer can contribute to the formation of a unique system of transport channels. Incorporation of HAS facilitated the formation of supramolecular clusters in PPO. This phenomenon was caused by intra- and intermolecular segregation of polar polymer chains, which created zones that assisted the selective migration of separated molecules inside membranes. An increase in the HAS content in the PPO matrix up to 5 wt.% led to an increase in the total flux (0.02 kg/m^2^·h) and a significant increase in the separation factor (11,240).

Nanosilica was used by Sabzevari et al. [77] to modify polyamide. The scanning electron microscopy data confirmed that agglomeration of nanosilica occurred only at relatively high contents of the filler. Various contents of nanosilica in the polyamide polymer were tested (0.5, 1, and 2 wt.%). MMM with 0.5 wt.% nanosilica was found to offer the best separation properties. The separation factor was significantly reduced at higher loads of nanosilica due to the agglomeration of nanoparticles in the polyamide matrix.

A summary of the performance data of the membranes described in this section can be found in Table 4.

**Figure 4:**
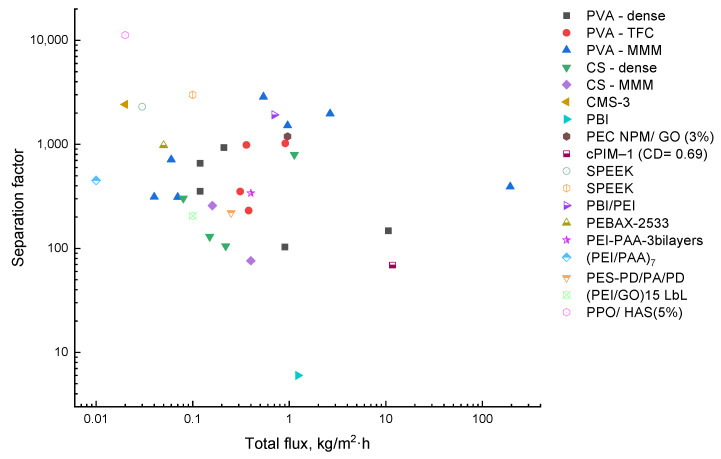
Comparison of the transport characteristics in the separation of water/EG mixture for the known membranes.

The PV performance of membranes in EG/water separation was systematically reported. Figure 4 shows the ratios between the total flux and the separation factor for all the membranes reviewed. The MMMs based on PVA established on the upper bond were shown to exhibit exceptional separation performance. However, the highest selectivity point was determined by PPO/HAS (5%). Unfortunately, this membrane possessed one of the lowest fluxes, which could be overcome by fabricating TFC membranes with a microporous support layer. CS membranes generally provided less favorable characteristics in EG dehydration. It should be emphasized that some hydrophobic polymers (SPEEK, PBI/PEI, PEC) offered good separation with both sufficient values of separation factor and flux.

## 5. Membranes for Methanol/EG Separation

The combined use of methanol and ethylene glycol in certain syntheses and industrial processes necessitates the regeneration and utilization of alcohols. Unlike conventional distillation, pervaporation is a low-energy-consuming technology for removing methanol from EG solutions.

However, pervaporation separation of organic mixtures is not so advanced as dehydration, despite the great industrial importance of this process. Methanol and EG have almost similar polarities, although the difference in their molecular weight and size is significant. Therefore, PV studies of methanol/ethylene glycol systems appear to be a fundamental challenge, since the results of the analysis may shed some light on the relative importance of the sorption and diffusion stages during mass transfer of molecules. Thus, the desired membrane should provide high selective sorption of methanol and selective methanol diffusion due to the larger molecular size of ethylene glycol. The important aspects of this problem are summarized in this section. According to the literature data, both hydrophilic and hydrophobic materials show high methanol selectivity.

A great number of studies were devoted to poly(2,6-dimethyl-1,4-phenylene oxide) (PPO) as a membrane material, because this polymer is chemically resistant and possesses good mechanical and thermal properties. PPO separation performance has already been investigated by many authors in the gas separation and pervaporation processes. Thus, Khayet et al. [78] applied PPO membranes in the separation of methanol–EG mixtures over the entire concentration range. The effect of the nature of casting solvents on the PPO membrane morphology, surface characteristics, and transport properties was estimated. The authors also used the Flory–Huggins theory to predict solubility parameters and explain sorption behavior of the membranes. It was found that PPO membranes prepared with the use of chloroform (PPO–CH) showed better pervaporation performance than the films cast from solutions in 1,1,2-trichloroethylene (PPO-TC), mainly due to higher diffusion selectivity. Meanwhile, varying the solvent nature had practically no impact on the surface properties and the solubility parameters of the two PPO membranes. The calculated parameters of interaction between methanol and PPO were about twofold lower than those found for the EG/PPO interaction. This fact, along with higher diffusion capability of methanol through PPO matrix, contributed to preferred methanol transport through both PPO–CH and PPO-TC. The typical tradeoff dependence between total permeation flux and selectivity was also observed in this study. The authors noted that the developed membranes had higher methanol selectivity at almost similar permeability compared to the cellophane membrane [5] discussed above.

The use of mixed matrix polymer materials is another approach to the development of novel membranes. Hence, a number of studies carried out by Polotskaya et al. [79,80,81,82] were devoted to the development of MMMs based on PPO containing hybrid macromolecules as fillers, as well as to a further investigation of their performance in the pervaporation of methanol/EG mixture.

In particular, MMMs were obtained by inclusion of hybrid star-shaped macromolecules (HSMs) (up to 5 wt.%) into the PPO matrix. HSM consisted of polar poly(2-vinyl-pyridine)-*block*-poly(*tert*-butylmetacrylate) (P2VP-*block*-PTBMA) and nonpolar polystyrene (PS) arms grafted onto fullerene C_60_ branching center [79]. This choice of modifying additive was explained as follows: the arms of different polarities provided the compatibility between the PPO matrix and components of the star modifier, which promoted the uniform distribution of HSM within the PPO matrix. The authors revealed significant morphological changes in the obtained membranes compared to the pure PPO films; PPO/HSM membranes contained domain structures of rounded shape, and their size increased with the growth of the HSM content. These structural features of PPO/HSM provided higher values of swelling degree and diffusion coefficient of the separated liquids (in comparison with those of pure PPO membranes). All the membranes under study were selective to methanol; however, the best separation performance was achieved for the membrane containing 5 wt.% HSM (the flux was equal to 0.08 kg/m^2^·h, and the separation factor was equal to 930 for the feed containing 5 wt.% methanol). The structure of PPO/HSM membranes was characterized by small-angle neutron scattering and scanning electron microscopy [80]. The results revealed qualitative differences between geometries of diffusion channels in the PPO matrix and the modified PPO/HSM membranes exposed to swelling in deuterated methanol. The effect of HSM filler on the transport properties of PPO when separating the methanol/EG mixture was explained in terms of changes in polymer chain packing. The authors suggested that star-shaped macromolecules stimulated the transformation of branched system of transport channels in the polymer matrix into linear channels. This facilitated the transport of the separated liquids through the PPO/HSP membranes, as established in [79].

In order to understand the effect of the polymer star structure on properties of hybrid membranes, Polotskaya et al. [81] and Vinogradova et al. [82] investigated the simplest model polymer stars (fullerene-containing polystyrene (FPS) that had only six arms of nonpolar PS). The FPS was added to PPO matrix in various amounts (up to 5 wt.%), and the effect of FPS presence, feed composition, and operating temperature on the PPO/FPS separation performance was studied. The investigation of membrane morphology revealed that the PPO/FPS (5%) membrane had a more compact structure than unmodified PPO, which was consistent with the data on membrane density (1.0398 g·°C·cm^−3^ for PPO and 1.0462 g·°C·cm^−3^ for PPO/FPS (5%)). Sorption–desorption experiments carried out for methanol and EG in PPO/FPS membranes showed that an increase in FPS content in the PPO matrix resulted in decreasing sorption degrees and diffusion coefficients of both alcohols. Meanwhile, the sorption degree and diffusion coefficient for methanol were several times higher than those for EG, which facilitated the selective transport of methanol through PPO/FPS. These results agreed with previously reported findings [79,80]. An increase in operating temperature (from 20 to 60 °C) and in methanol concentration in the feed (from 10 to 30 wt.%) led to a decrease in separation factor and total flux for all the membranes. Compared to PPO/HSM (5%) studied in [79], PPO/FPS (5%) had lower values of total flux and separation factor, indicating a significant effect of polar P2VP-*block*-PTBMA arms on mass transfer through the hybrid membrane.

Chitosan membranes were also successfully applied in the removal of methanol from organics. However, the main constraint of chitosan-based materials is a loss of both the permselectivity and mechanical strength due to considerable swelling in alcohol solutions. Several attempts have been made to overcome this issue. Zhang et al. [83] applied poly(*N*-vinyl-2-pyrrolidone) (PVP) and UV radiation to prepare UV-crosslinked chitosan/PVP blended membranes. The obtained dense films were tested in the pervaporation of a methanol/EG mixture. The authors reported that UV irradiation of the polymer blend solution allowed them to obtain a highly crosslinked polymer network within the membrane structure. Compared to pristine chitosan films, the crosslinked CS/PVP blended membranes had higher tensile strengths and thermal stability. Moreover, the authors noted that an increase in PVP content in the membrane led to an increase in permeability when separating the methanol/EG mixture, while an increase in irradiation time led to much higher values of separation factor. The optimal values of PVP content and UV irradiation time were about 9 wt.% and 4 min, respectively, which provided the highest membrane permselectivity. Several studies by Zhang et al. [84,85] were devoted to the development of MMMs based on chitosan/PVP blends modified by silica. The hybrid membranes were obtained via a sol–gel process using 2-bis(triethoxysilyl)-ethane (BTEE) as a crosslinker that formed a semi-interpenetrating network in the membrane matrix at the molecular scale [84]. According to the authors, this approach was intended to suppress membrane swelling and enhance methanol selectivity. PVP was added in order to improve permeation fluxes. The key results of the research were as follows: the optimal chitosan/PVP ratio in the membranes was 10, which provided the highest pervaporation separation index (PSI) of 35.8; addition of BTEE (up to 10.4 wt.%) into the polymer blend allowed the authors to improve the mechanical stability of the membranes, to decrease the surface hydrophilicity, and to change the membrane structure. The membrane became denser than pure chitosan and chitosan/PVP blended membranes, because a highly cross-linked network was formed within the polymer matrix. This had a positive effect on permselectivity of the MMMs. The hybrid membrane containing 10.4 wt.% BTEE had the highest separation factor of 6129 in the separation of the methanol–EG mixture (6:94 wt.%) at 60 °C.

In their further research [85], these authors used tetraethoxysilane (TEOS) instead of BTEE as an additive to form the semi-interpenetrating network structure in the membrane. The effects of PVP and TEOS contents on the physicochemical properties and pervaporation performance of the developed membranes were studied in detail. In the case of the CS/PVP–TEOS hybrid membranes, the authors observed a trend similar to that reported in [84]; addition of TEOS led to lower crystallinity, higher thermal stability, and a denser structure compared to the pristine CS and CS/PVP membranes. The sorption selectivity of the MMMs toward methanol and the separation factor also increased with TEOS content, while the permeation flux decreased. The highest separation factor (5579) and a PSI of about 300 were achieved for the MMM with 6.51 wt.% PVP and 28.40 wt.% TEOS at 60 °C.

PIMs represents a relatively novel type of membrane materials that have attracted attention due to their excellent chemical and physical resistance and high surface area values. PIMs have already been employed as membrane materials for gas separation and pervaporation, particularly for the removal of methanol from its mixtures with EG. Wu et al. [86] investigated the effect of feed composition and temperature on the performance of dense PIM-1 membranes in the separation of water/EG and methanol/EG mixtures. The authors found that the membranes were preferably permeable toward methanol as a result of the lower size and activation energy of methanol molecules compared to EG. The total flux increased with increasing methanol concentration in the feed and temperature. The highest value of total flux was about 24 kg·μm/m^2^·h. However, the obtained membranes showed rather low values of separation factor (the highest one was about 30 at 20 °C).

Ghosh et al. [87] conducted extensive research on commercial cellophane films for the separation of methanol/EG mixture under different feed temperatures and compositions. In order to improve separation properties of cellophane, the authors immersed cellophane films into the individual separated liquids (methanol and EG) for 24 h at heating up to 40 °C (so-called solvent-annealing technique). It was found that this membrane conditioning had a certain effect on membrane morphology and caused changes in the diffusion-sorption capacity and liquid–polymer interactions in the membrane. All the studied membranes were selective toward methanol. The highest overall separation performance at low methanol content in the feed (below 32 wt.%) was achieved for the membrane treated with EG at 30 °C (CEG-30). The authors attributed this fact to the optimum plasticization degree of CEG-30 as compared with CEG-40, CM-30, and untreated membrane, which resulted in higher diffusion coefficients and permeation rates of methanol in the case of CEG-30. This study showed that pretreatment of membrane using the solvent-annealing technique could be a promising way of improving membrane transport properties in certain cases.

Ray et al. [88] developed three types of methanol-selective membranes based on copolymers of acrylonitrile (AN) with hydroxy ethyl methacrylate (HEMA), methacrylic acid (MAC), and vinyl pyrrolidone (VP) of different compositions. The use of copolymers makes it possible to combine valuable characteristics of each monomer within a single membrane material. Thus, Ray and coauthors suggested that HEMA and MAC, whose solubility parameters are close to those of methanol, would increase permeability, while AN would maintain the integrity of the membrane. It was found that the sorption degree of methanol was slightly higher than that of EG, whilst the diffusion rate of methanol far exceeded that of EG for all the developed membranes. It was also demonstrated that sorption and diffusion selectivity toward methanol decreased in the order PANHEMA > PANMAC > PANVP. As a result, the permeation selectivity of methanol was much higher than that of EG and followed the same order: PANHEMA > PANMAC > PANVP. The authors reported good selectivity for the PANHEMA and PANMAC membranes (separation factors of 14.74 and 11.3, respectively), but moderate methanol flux (kg/m^2^·h and 0.0813 kg/m^2^·h, respectively) in the separation of the feed mixture containing 50 wt.% methanol.

The parameters of pervaporation performance of the polymer membranes reviewed in this section are listed in Table 5.

**Figure 5:**
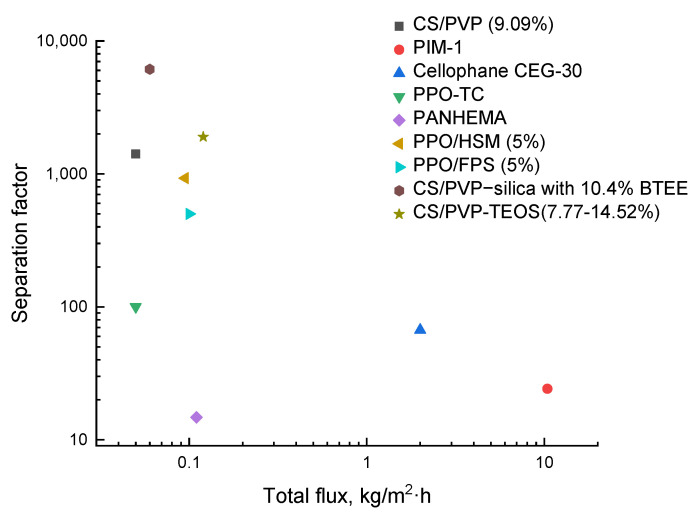
Comparison of the transport characteristics in the separation of methanol/EG mixture for the known membranes.

The relationship between separation factor and total flux for methanol/EG separation is illustrated in Figure 5. As seen in the presented diagram, the CS/PVP–silica membranes showed the highest separation selectivity but relatively low permeability. On the contrary, the PIM-1-based membranes had the highest permeability and low separation selectivity. It should be noted that the membranes based on CS/PVP blend with inorganic modifiers are specifically promising for removing methanol from its mixtures.

## 6. Conclusions and Future Outlook

This review presented the research devoted to the development and investigation of pervaporation membranes for the purification of EG from water and methanol impurities. In view of the constantly expanding field of EG applications, the search and implementation of novel economical and environmentally friendly technologies for the separation of organic and aqueous–organic solutions are still an issue. To date, the research in the field of pervaporation has focused on membranes with high selectivity and long-term stability, which would make them suitable for industrial applications. This review was dedicated to investigating the influence of various nature and structure of polymer membranes on PV performance. Analysis of the literature data led us to conclude that controlled changes in the structure and transport properties of a membrane can be made using modification methods such as treatment with organic solvents, introduction of nonvolatile additives, polymer blending, crosslinking, and heat treatment. The creation of mixed matrix membranes and use of various modifiers have a particularly positive effect on the separation selectivity of membranes.

As discussed above, only a few of membranes showed promising performance in separating EG from impurities. The MMMs based on PVA provided the most optimal properties for EG dehydration [46,52]. The CS-based membranes exhibited exceptional pervaporation performance for methanol/EG mixtures [86]. However, they are still far from being suitable for industrial use. Currently, there are two main research trends in the field of large-scale applications. Firstly, these are attempts to obtain high-performance, stable, and cost-effective membranes using various modification methods. An effective strategy is the introduction of various hybrid fillers into polymer matrices, which allows improving operational stability and overcoming permeability–selectivity tradeoff relations.

Secondly, more attention should be paid to the possibility of integration between reaction and separation systems. Such a solution could significantly increase the reaction yield and selectivity, for example, in the synthesis of EG or PET. In this case, membrane performance would play a key role in the selection of membrane materials, along with increased long-term operational stability including selectivity, chemical and temperature resistance, and durability. Much effort should be made to develop thin-film composite and dual-layer membranes, which will maintain the high selectivity of the process and reduce the effective membrane area without performance deterioration.

In general, the analysis of the studies devoted to use of pervaporation membranes in the purification of EG after synthesis or EG used as a reagent in various processes allows us to expect further success in this area. Since the pervaporation mechanism involves selective sorption (one of limiting stages), the most efficient approach consists of the use of hydrophilic membranes, such as PVA and chitosan, for dehydration of EG and removal of polar methanol. TFC membranes based on PVA provided a moderate separation factor and excellent total flux (391 and 194 kg/m^2^·h). However, it should be noted that hydrophobic membranes are capable of exhibiting much higher selectivity due to diffusion limitations associated with differences in molecular sizes. The PPO/HAS membrane showed deficient flux and a good separation factor (0.02 kg/m^2^·h and 11,240) in EG dehydration, which is much higher than recorded for the majority of reported membranes. Therefore, both types of membranes are complementary for the tasks under consideration.

Although a large number of various membranes have already been developed, there is still room for improvement of permeation flux and selectivity. In addition, many factors, including the cost of membrane preparation and configuration of the membrane module, prevent their use on an industrial scale. Further studies of PVA and polyimide membranes aimed at controlling their chemical structure and the introduction of new precursors are recommended to attain the sustainable development of these membranes. Since synthetic methods are constantly being improved, and new materials are being developed (e.g., star-shaped macromolecules, MOFs, polyelectrolytes), we may expect that future research focused on long-term stability will offer new opportunities for the production of membrane modules, on both laboratory and industrial scales.

## Figures and Tables

**Table 1:** Physical properties of EG [5,17].

Property	
Molecular weight	62 g/mol
Boiling point at 760 mmHg	197.6 °C
Density at 40 °C	1099 kg/m^3^
Viscosity at 20 °C	19.8 × 10^−3^ Pa·s
Normal freezing point	−13.0 °C
Vapor pressure at 20 °C	7.5 Pa
Flash point, Cleveland open-cup method	115.0 °C
Surface tension at 25 °C	48.0 × 10^−3^ N/m
Water solubility at 20 °C	100%
Critical specific volume	19.1 × 10^−2^ L·mol/g

**Table 2:** Performance of PVA-based membranes for the separation of water/EG.

Polymer	EG in Feed, wt.%	T, °C	Membrane Performance		Ref
			Separation Factor	Total Flux, kg/m^2^·h	
PVA	90	60	-	66.70	[24]
FIPN50	97.1	75	148	10.63	[28]
PAA/PVA = 30/70	90	30	103	0.90	[29]
CS–PVA2	90	25	659	0.12	[30]
PVA	90	25	354	0.12	[30]
PVA(GA15)	80	70	933	0.21	[31]
PVA–PES 0.2% Borax	80	45	3.5	6 m^3^ (stp)/m^2^·h	[32]
PVA/PP	80	60	1021	0.91	[33]
PVA/PES (0.5 wt.% borax)	80	70	352	0.31	[34]
PVA–PES	82.5	80	231	0.38	[35]
PVA4 (TMC)	90	60	987	0.36	[36]
PVA/NaA (5%)	80	70	1520	0.96	[44]
PVA/NaA	90	60	-	0.35	[45]
PVA/PP/Zeolite 4A (5%)	80	70	1972	2.65	[46]
PVAm–PVA on PSf support/ CNT (0.5)	97	70	391	194.00	[47]
PVA/MPTMS 50	80	70	311	0.07	[48]
PVA–GPTMS/TEOS 1:1	80	70	714	0.06	[49]
PVA10 wt.% HBPE	90	25	312	0.04	[50]
PVA (SO3H-MIL-101-Cr)	90	70	2864	0.54	[52]

**Table 3:** Performance of chitosan-based membranes in EG dehydration.

Polymer	EG in Feed, wt.%	T, °C	Membrane Performance		Ref
			Separation Factor	Total Flux, kg/m^2^·h	
CS	90	30	129.5	0.15	[55]
M-CA/CS	96.8	30	302	0.08	[56]
Surface crosslinked CS/PES	80	80	796	1.13	[57]
PECM60/40	80	70	105	0.22	[58]
Chitosan–poly(acrylic acid) polyelectrolyte complex/mordenite	80	70	258	0.16	[59]
Chitosan-coated zeolite-filled cellulose membrane	95	30	76	0.4	[60]

**Table 4:** Performance of mixed matrix membranes in EG dehydration.

Polymer	EG in Feed, wt.%	T, °C	Membrane Performance		Ref
			Separation Factor	Total Flux, kg/m^2^·h	
CMS-3	95	30	2419	0.02	[61]
cPIM–1 (CD = 0.69)	80	30	69	11.73	[62]
SPEEK	90	32	2300	0.03	[6]
SPEEK	90	30	2991	0.10	[63]
PBI	50	25	6	1.23	[64]
PBI/PEI	80	50	1925	0.70	[65]
PEBAX-2533	94.7	30	978	0.05	[66]
PEI–PAA 3 bilayers	97	40	340	0.40	[67]
(PEI/PAA)_7_	95	22	450	0.01	[68]
PDMAEMA/PSF	99.7	30	32,901	1 mol/(m^2^·h)	[69]
PES–PD/PA/PD	89.5	38	220	0.25	[70]
PEC NPM/GO (3%)	90	60	1191	0.96	[71]
(PEI/GO) 15 LbL	95	35	205	0.10	[72]
PPO/ HAS (5%)	90	50	11,240	0.02	[76]

**Table 5:** Performance of the membranes for methanol/EG pervaporation.

Polymer	EG in Feed, wt.%	T, °C	Membrane Performance		Ref
			Separation Factor	Total Flux, kg/m^2^·h	
CS/PVP (9.09%)	94	25	1410	0.05	[83]
PIM-1	71.5	30	24.2	10.40	[86]
Cellophane CEG-30	85	30	67	2.00	[87]
PPO-TC	90	30	100	0.05	[78]
PANHEMA	50	30	14.74	0.11	[88]
PPO/HSM (5%)	95	50	930	0.095	[79,80]
PPO/FPS (5%)	95	50	500	0.10	[81]
CS/PVP−silica hybrid membrane with 10.4% BTEE	94	60	6129	0.06	[84]
CS/PVP–TEOS (7.77–14.52%)	94	60	1899	0.12	[85]

## Abbreviations

The following abbreviations are used in this manuscript:

AA	acrylic acid
AM	acrylamide
AN	acrylonitrile
ATR-FTIR	Fourier-transform infrared spectroscopy with attenuated total reflectance
bis-MPA	2,2-bis (hydroxymethyl) propionic acid
BTEE	2-bis(triethoxysilyl)-ethane
CA	calcium alginate
CMS-3	perfluoropolymer
CNT	carbon nanotube
cPIM-1	carboxylated PIM-1
CS	chitosan
DMO	dimethyl oxalate
DSC	differential scanning microscopy
EG	ethylene glycol
EO	ethylene oxide
FIPN	full interpenetrating network
FPS	fullerene-containing polystyrene
GA	glutaraldehyde
GO	graphene oxide
GPTMS	γ-glycidyloxypropyltrimethoxysilane
HAS	heteroarm star
HBPE	hyperbranched polyester
HEMA	hydroxy ethyl methacrylate
HSM	hybrid star-shaped macromolecules
IPN	interpenetrating network
MAC	methacrylic acid
MBA	methylene-bis-acrylamide
MMM	mixed matrix membrane
MOF	metal–organic frameworks
MPTMS	γ-mercaptopropyltrimethoxysilane
*p*-TSA	*p*-toluenesulfonic acid
PA	polyamide
PAA	polyacrylic acid
PAAM	polyacrylamide
PBI	polybenzimidazole
PD	polydopamine
PDMAEMA	poly(*N*,*N*-dimethylaminoethyl methacrylate)
Pebax	poly(ether-*block*-amide)
PEC NP	polyelectrolyte complex nanoparticle
PECM	polyelectrolyte complex membrane
PEI	polyetherimide
PEIm	polyethyleneimine
PES	polyethersulfone
PET	polyethylene terephthalate
PIM	polymers with intrinsic microporosity
PP	polypropylene
PPO	poly(2,6-dimethyl-1,4-phenylene oxide)
PS	polystyrene
PSF	polysulfone
PTBMA	poly(*tert*-butyl methacrylate)
PV	pervaporation
PVA	poly(vinyl alcohol)
PVDF	polyvinylidene fluoride
P2VP	poly(*N*-vinyl-2-pyrrolidone)
PVSM	polyvinylamine
SPEEK	sulfonated poly(ether ether ketone)
TEOS	tetraethoxysilane
TFC	thin-film composite
TMP	1,1,1-trimethylolpropane
TMS	trimesoyl chloride
VP	vinyl pyrrolidone
XRD	X-ray diffraction
**Symbols and Units**	
*J*	total flux (kg/m^2^·h)
*M*	weight of permeate (kg)
*S*	membrane area (m^2^)
*t*	test period (h)
T	temperature (°C)
x	weight percentage of components in the feed
y	weight percentage of components in the feed
*β*	separation factor
